# Born Too Soon: Women’s health and maternal care services, seizing missed opportunities to prevent and manage preterm birth

**DOI:** 10.1186/s12978-025-02034-w

**Published:** 2025-06-23

**Authors:** Bo Jacobsson, Jennifer Harris Requejo, Teesta Dey, Tina Lavin, Margaret Titty Mannah, Ramkumar Menon, Catalina Valencia, Gaurav Sharma, Andrew Shennan, Elham Shakibazadeh, Pisake Lumbiganon, Sarah Bar-Zeev, Sue Steen, Angela Nguku, Hiromi Obara, Lisa Noguchi, Pius Okong, Sanjukta Amrita Singh, Jane Sandall, Anna Gruending, Zahida Qureshi, Joshua P. Vogel

**Affiliations:** 1 University of Gothenburg, Gothenburg, Sweden; 2 World Bank, Washington, DC, USA; 3 Development and Research Training in Human Reproduction (HRP), Department of Sexual and Reproductive Health and Research, UNDP/UNFPAUNICEF/WHO/World Bank Special Programme of ResearchWorld Health Organization, Geneva, Switzerland; 4 Ministry of Health, Freetown, Sierra Leone; 5 Department of Obstetrics and Gynecology, Director, Division of Basic Science and Translational Research, The University of Texas Medical Branch at Galveston, Galveston, USA; 6 Maternal Fetal Medicine Unit Clinica del Prado - University CES, Medellín, Colombia; 7 Jhpiego - Johns Hopkins University, Washington, DC USA; 8 King’s College London, London, UK; 9 Department of Health Education and Promotion, School of Public Health, Tehran University of Medical Sciences, Tehran, Iran; 10 Department of Obstetrics and Gynaecology, Faculty of Medicine, Khon Kaen University, Khon Kaen, Thailand; 11 United Nations Population Fund UNFPA, NY HQ/Burnet Institute, Melbourne, Australia; 12 Birth With Dignity, Uganda & USA/Maple Grove Hospital, Maple Grove, MN USA; 13 White Ribbon Alliance Kenya As the Founder and Lead and White Ribbon Alliance Global Movement As the Co-Convener, Nairobi, Kenya; 14 Bureau of International Health Cooperation, National Center for Global Health and Medicine, Shinjuku, Japan; 15 FIGO HSS & RC Committee, MK Post Graduate School Uganda Martyrs University, Nkozi, Uganda; 16 The London School of Hygiene & Tropical Medicine, LSHTM, London, UK; 17 Department of Women and Children’s Health, King’s College, London, UK; 18 Partnership of Maternal, Newborn, Child Health, Geneva, Switzerland; 19 Department of Obstetrics and Gynecology, University of Nairobi, Nairobi, Kenya; 20 Women’s, Children’s and Adolescents’ Health Program, Burnet Institute, Melbourne, Australia

**Keywords:** Preterm birth, Quality of care, Prevention, Stillbirth, Sexual reproductive and maternal health services

## Abstract

**Progress:**

The past ten years have seen uneven developments in women's and adolescents' health and reproductive rights. Globally, reductions of maternal and neonatal mortality rates and adolescent birth rates have been achieved along with improvements in coverage of key reproductive and maternal health services. However, preterm birth rates have not changed significantly. There is still large variation in these rates across the world, with the highest rates occurring in South Asia and sub-Saharan Africa.

**Programmatic priorities:**

Effective interventions based on current clinical guidelines are available that can prevent preterm birth or reduce its negative impacts on newborns. These recommended interventions can be delivered as part of essential health service packages during the preconception, antenatal, intrapartum, and postnatal phases. They encompass comprehensive family planning services that enable women and adolescent girls to determine the timing and number of children they have, and the provision of prevention and treatment-related interventions during pregnancy, childbirth, and the postnatal period that improve maternal and newborn health including reducing preterm births as well as stillbirths. Health system improvements are needed so that all women are reached with these services and that they are provided respectfully and according to standards.

**Pivots:**

To better prevent and manage preterm births as part of broader goals of improving maternal and newborn health, health systems need to be strengthened so that all women are reached with essential packages of care before, during, and after pregnancy and childbirth. Achieving this and integrating these service packages into universal health coverage strategies requires collaboration across government leaders, civil society members, private sector actors, and development partners. Increasing coverage of antenatal care, institutional delivery, and postnatal care represents an opportunity to improve the quality of care provided during those service contacts including through the provision of interventions that address modifiable risk factors for preterm birth such as prevention and treatment of infections, poor nutritional status, and substance use. Other pivots to enhance the quality of care include using existing tools to optimize the management of preterm birth, such as appropriate use of antenatal corticosteroids, and providing respectful person-centred care for women, adolescents, and families.

## Plain language summary

Over the past decade, progress in women's and adolescents' health and reproductive rights has been uneven. Significant advancements have been made in family planning, antenatal care, skilled birth attendance, postnatal care, and the provision of sexual and reproductive health services. Maternal and neonatal mortality rates have dropped, although the pace of decline has slowed in recent years. Preterm birth rates, however, have remained relatively unchanged across regions, with most preterm births occurring in low- and middle-income countries.

Programmatic priorities focus on the health sector's ability to offer evidence-based services to all women that can prevent preterm birth or mitigate the effects on newborns. This can be achieved through the implementation of high-quality service packages during the preconception, antenatal, intrapartum, and postnatal stages, based on current clinical guidelines. Also, well-coordinated intersectoral interventions are needed for countries to achieve substantial preterm birth reductions.

Health systems must be strengthened so that they are ready to provide high quality services for the prevention and management of preterm birth, most of which are also effective at improving other aspects of maternal and newborn health including prevention of stillbirths. This requires collaboration among government leaders, civil society, the private sector, and development partners. Integrating sexual, reproductive, and maternal health services into primary health care systems and working towards universal health coverage are critical steps in achieving these goals.

To reduce preterm births, service packages that improve women's nutrition, prevent infections and reduce stress and substance use during pregnancy should be scaled up. Additionally, enhancing the quality of care for women and adolescents before, during, and after childbirth is crucial. This includes optimizing preterm birth management with antenatal corticosteroids and providing respectful, person-centered care that values women's voices in the health care context.

## Key findings

### Progress

- The coverage of health services for women has improved globally in most countries and focus is now needed also on quality and experience of care.
- Unacceptable inequities in coverage and quality remain and hinder progress towards the effective prevention and management of preterm birth, and on reducing the number of stillbirths.

### Programmatic priorities

- Women’s access to a comprehensive set of high-quality, respectful services for sexual, reproductive and maternal health is fundamental to improving health outcomes, including the prevention and care of preterm birth, and to achieving universal health coverage.
- Preconception care, including ensuring that all women and adolescent girls can determine the number and spacing of their children.
- Pregnancy: evidence-based, high-quality, and respectful antenatal care to reduce the likelihood of preterm birth occurring and to improve maternal and newborn health outcomes more broadly.
- Childbirth: evidence-based, high-quality and respectful care around the time of childbirth. When preterm birth is imminent before 34 weeks’ gestation and adequate childbirth and preterm newborn care is available, antenatal corticosteroids should be used to prevent newborn morbidity and mortality.
- Postnatal care: evidence-based, high-quality and respectful postnatal care to facilitate positive health outcomes for the woman, the newborn and the family. In the case of stillbirth or neonatal death, it is vital to ensure that women and their families are offered compassionate bereavement care.

### Pivots

- Emphasize that government, civil society, the private sector and all development partners must join forces to ensure the effective integration of sexual, reproductive and maternal health services within universal health coverage, as well as integrating action on the known modifiable risk factors for preterm birth.
- Seize the opportunity of recent increases in coverage of women’s sexual, reproductive and maternal health care to improve the quality of care before, during and after childbirth, provided by multidisciplinary teams of health-care providers in partnership with women.

## Introduction

This paper is part of the Born Too Soon supplement and focuses on opportunities to strengthen health systems and reach all women with services proven effective at reducing preterm birth and in improving their own health.

The papers in this supplement were developed from the report “*Born Too Soon: A decade of action on preterm birth”* [1]. The report was part of a campaign to create a movement for preterm birth, linked to the need to accelerate progress for maternal and newborn health and preventing stillbirths, noting slowing of momentum, with flatlining progress for preterm birth being foundational. Content derives from evidence synthesis of new data, literature reviews and case studies highlighting policy, implementation and community perspectives, collated into three themes: (1) *progress* particularly in the last decade; (2) programmatic priorities based on evidence; and (3) pivots needed to accelerate change in the decade ahead. The first paper in this series summarizes definitions and terminology [2]. Table 2 describes the three levels of preterm birth prevention and includes the definition of preterm birth and known risk factors [3, 4].

## Main body

### Progress

**Fig. 1:**
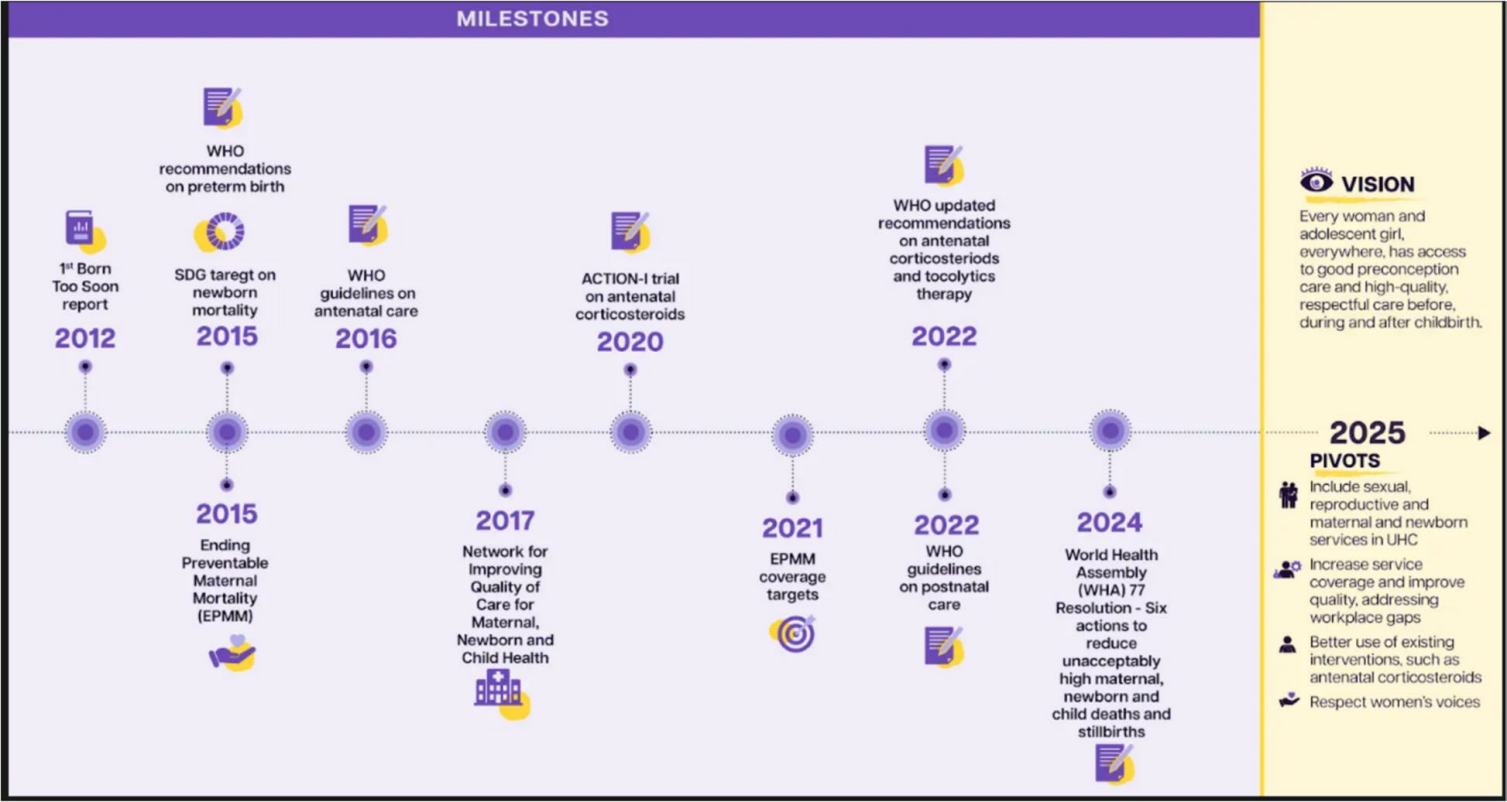
Maternal health and preterm birth: Timeline of progress in the past decade and a vision for the future

**Fig. 2:**
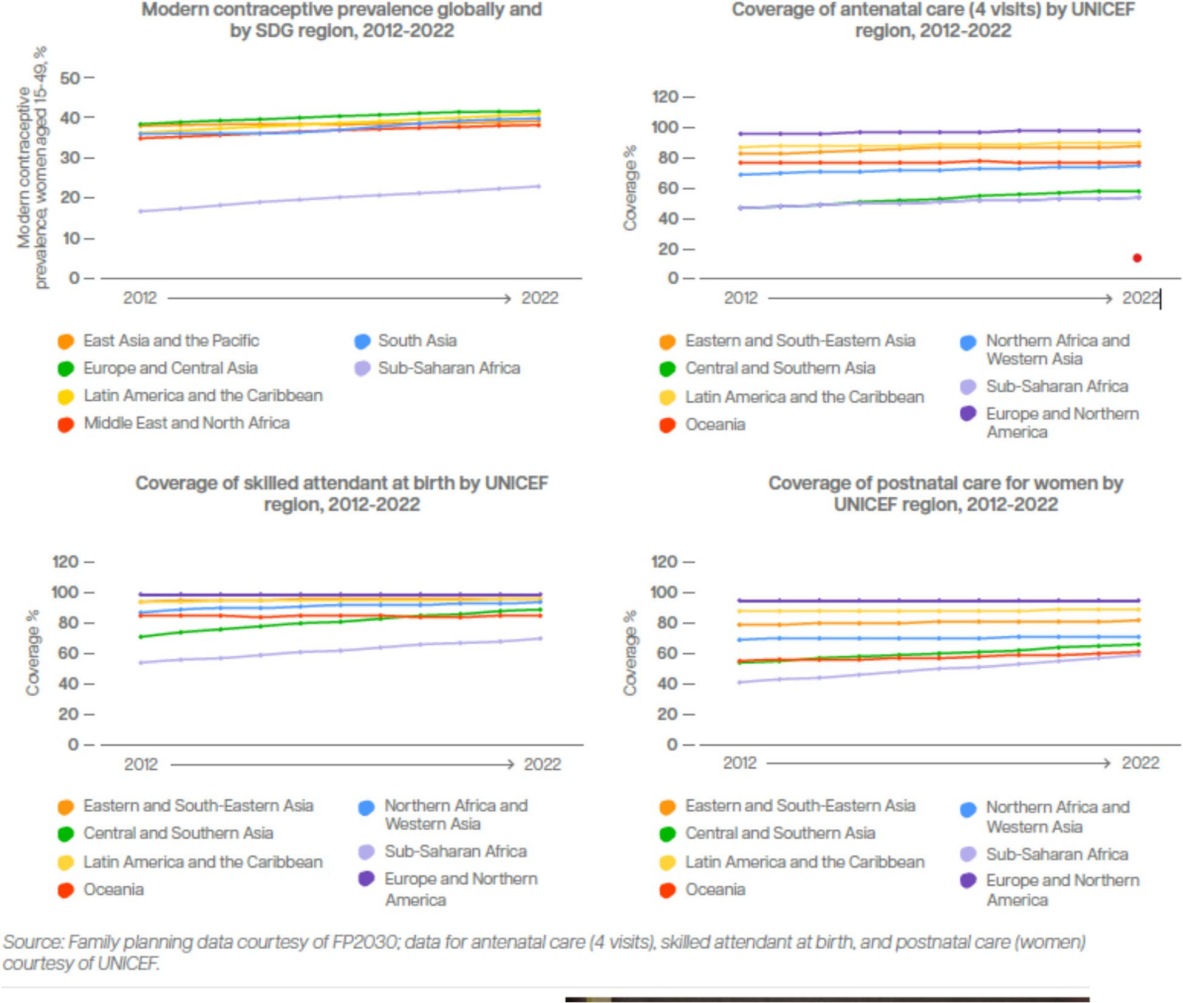
Coverage trends by region of essential sexual, reproductive, and maternal health services, 2012–2022

In the last decade, women’s and adolescents’ health and reproductive rights have advanced unevenly, with progress on some issues and in certain countries, and stagnation and rollbacks in others [5, 6]. Positive developments over the past ten years were bolstered by new evidence including the introduction of new health policies and technical guidelines such as those on preterm birth prevention and management (Fig. 1). Improvements in the coverage of sexual, reproductive and maternal health services have also been achieved in many countries across all income levels [5]. Fig. 2 shows progress in coverage of four lifesaving interventions that benefit both women and newborns: met need for family planning, antenatal care (four visits), skilled attendant at birth and postnatal care. In addition, the adolescent fertility rate in low- and middle-income countries (LMICs) dropped from 52 per 1000 women aged 15–19 years in 2012 to 43 in 2020 [7].

Growing recognition that increasing intervention coverage alone is insufficient for achieving mortality reductions has resulted in a greater focus on the quality of care in the Sustainable Development Goal (SDG) era [8]. In 2017, for example, 10 countries, supported by the World Health Organization (WHO), the United Nations Children’s Fund (UNICEF), and the United Nations Population Fund (UNFPA), joined forces to establish the Network for Improving Quality of Care for Maternal, Newborn and Child Health aimed at ensuring every woman, child, and adolescent receive high quality care throughout their life course [9]. A growing body of research has highlighted the benefits of women’s empowerment in improving access to vital services across the sexual, reproductive, maternal health spectrum [10, 11]. This literature, coupled with increased attention to quality of care, underpins a paradigm shift towards woman-centric models of care that promote women’s social autonomy and decision-making authority [13, 14]. Evidence shows that these approaches are key to the provision of respectful, high-quality care that delivers positive experiences for women before, during and after childbirth, and are emphasized in multiple WHO guidelines [15, 16].

Although there has been a pushback against sexual and reproductive health and rights in some countries, social movements in others have been engines of progress in advancing service access [18]. The “Green Wave” movement driven by collective action, for example, catalysed the liberalization of strict abortion laws in several Latin American countries [19]. In 2020, Argentina legalized abortion, followed by Colombia in 2022. In 2021, Ecuador introduced an exemption to the abortion ban for cases of rape, and in the same year, the Supreme Court of Mexico recognized a constitutional right to safe, legal and free abortion in early pregnancy, although access varies between states [19].

Despite these and other successes, much work remains. The latest United Nations maternal mortality estimates are a stark reminder that improving women’s health and well-being is an urgent and unfinished task [6]. While global maternal deaths reduced substantially between 2000 and 2015, such gains have largely stalled since 2015 and have even reversed in some countries. In 2020, there were an estimated 287 000 maternal deaths worldwide, 95% of which occurred in low- and middle-income countries. Between 2016 and 2020, only 31 countries achieved substantial reductions in maternal mortality, whereas 133 saw progress stall and 17, mostly in Europe and the Americas, experienced an increase [6]. Although global stillbirth rates declined about 35% in the past two decades, around 1.9 million stillbirths occurred in 2021. Around three-quarters of these stillbirths occurred in sub-Saharan Africa and Southern Asia, a reflection of glaring inequities in access to quality antenatal and delivery care services [20]. Stillbirths are a profound loss for the women and families that experience them and should be factored into women’s health strategies.

While there have been gains globally in coverage of reproductive, maternal and newborn services, major inequities in both coverage and quality of these services persist across and within countries and regions. Coverage of antenatal care (four visits) in Northern America and Europe, for example, is around 98% compared to 54% in sub-Saharan Africa. Within country disparities are equally glaring [21]. In Kenya, latest household survey estimates show that births attended by skilled health personnel range from 35% in the lowest wealth quintile to 92% in the highest [22]. Some of these disparities are linked to unaffordable care, “under the counter” user fees, infrastructure issues including insufficient numbers of facilities in remote and rural areas, and transportation bottlenecks (e.g., unreliable transport and poor road conditions) [23, 24].

In many places, increased coverage of sexual, reproductive, and maternal health services has not been accompanied by improvements in service quality due to various factors such as disrespectful care, insufficient numbers of trained providers and inadequate supplies of essential medicines and drugs. These inequities in service quality and coverage are a missed opportunity for the prevention and management of preterm birth, and more broadly for the prevention of stillbirths and maternal and newborn morbidity and mortality. More women may be attending antenatal care, but these visits may begin too late (after the first trimester) and not include screening for risk factors for preterm birth as well as for other maternal and newborn conditions [26]. Women often experience disrespect and abuse in many healthcare settings, which can dissuade them from utilizing needed health services in current pregnancies and the future [8, 27, 28]. External threats such as conflicts, disease outbreaks, and climate change also pose serious direct and indirect threats to women’s and adolescents’ health through exposures (e.g., pathogens, excessive heat, air pollution) and disruptions in transportation, health, nutrition, and social services. These same direct and indirect pathways also increase risks of preterm birth and stillbirths [2, 29, 30].

The evidence is clear: Promoting equitable access to respectful and high-quality sexual, reproductive, and maternal health services, including in resource-limited and fragile settings, is central to achieving meaningful improvements in maternal health and reductions in preterm birth and preterm-associated morbidity and mortality. Increased service access is also key to successful implementation of the World Health Assembly resolution adopted in May 2024 on accelerating progress towards achievement of the SDG targets on maternal, newborn, and child mortality. This resolution reflects renewed political commitment to ending preventable maternal and child deaths, including deaths due to preterm delivery.

The remainder of this paper describes what needs to be done to bridge the substantial gap between current scientific knowledge, and women’s and adolescent’s access to high-quality health services proven effective at preventing preterm births—as well as other causes of newborn deaths, stillbirths, and maternal deaths [32]. It is organized into two sections of programmatic priorities for the health sector and pivotal directions forward.

### Programmatic priorities

**Fig. 3:**
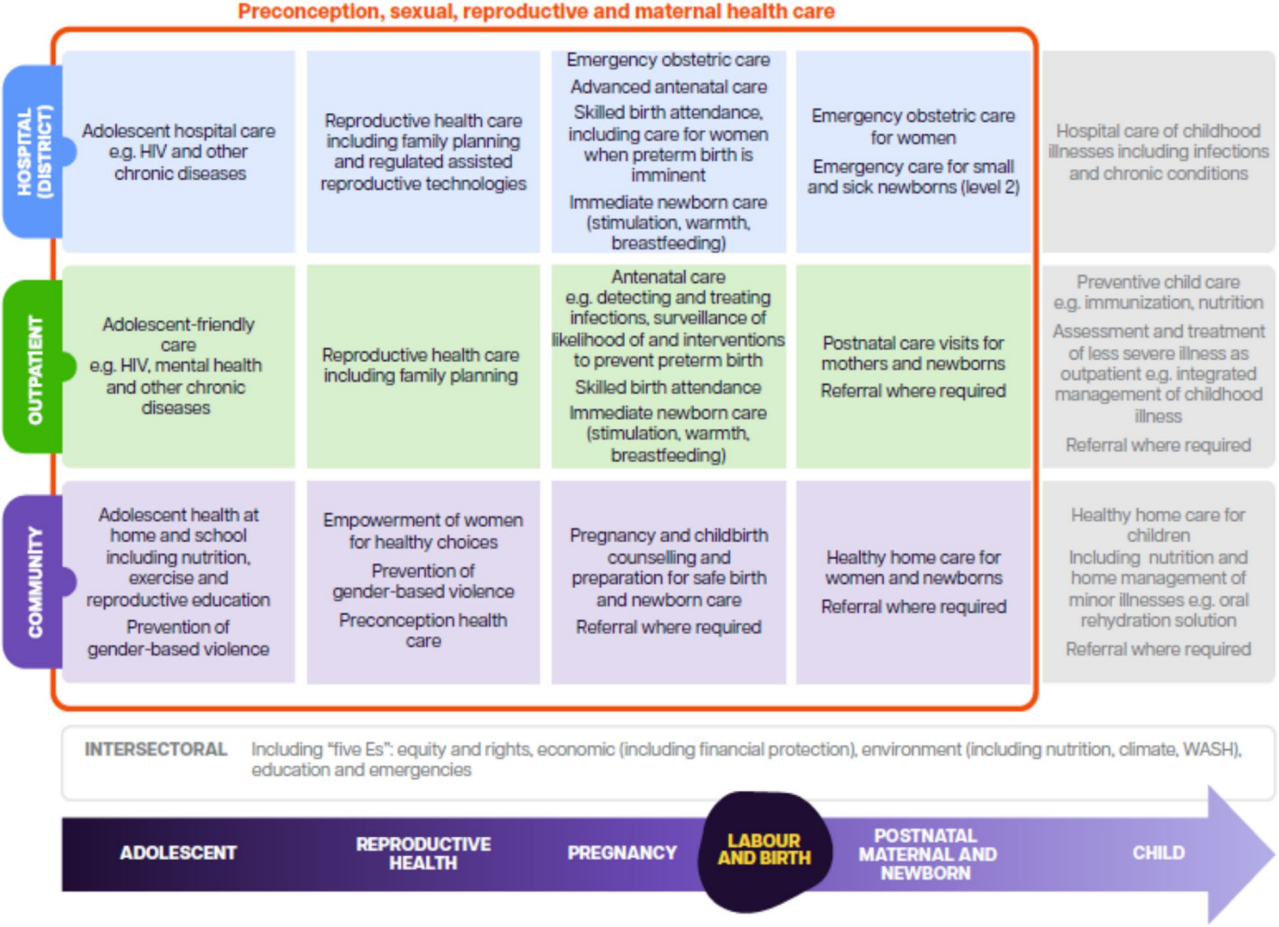
Health care packages for adolescent girls and women across the continuum of care

Embedded within the continuum of evidence-based sexual, reproductive, and maternal health services are specific interventions that can improve maternal outcomes, reduce stillbirths, and prevent preterm births or mitigate the effects of being born preterm (Fig. 3). (12, 13) These services and the key systems factors needed to achieve universal coverage of them with a high level of quality are discussed below [33, 34]. Intersectoral interventions that complement the health sector specific interventions are also needed to achieve substantial preterm birth reductions [36].

There are four high-impact essential packages to focus on as follows:

### Package 1: Preconception: a critical window for preterm birth prevention

Equitable access to high-quality sexual and reproductive health information and services are critical to ensure that every woman and adolescent girl can decide whether and when to get pregnant. These services also bring a wide range of social, economic and educational benefits with positive short- and long-term health effects and prevent many pregnancy-related health risks [16]. Despite setbacks in some countries, other countries have made considerable advances in implementing programmes designed to increase access to sexual and reproductive health services. Chile, for example, implemented an effective strategy to reduce adolescent pregnancies, with important knock-on effects for the prevention of preterm birth [37]. Early pregnancies negatively affect adolescent girls’ health and rights, and are associated with higher risks of preterm birth, low birth weight, and severe neonatal conditions. Recent evidence also shows that women born preterm are more likely to have a preterm birth themselves, showing how adolescent pregnancy contributes to an inter-generational cycle of risk, partly explained by genetic and non-genetic risk factors [38]. In response to its high adolescent fertility rate, Chile adopted the multi-sectoral regional Andean Plan for the Prevention of Adolescent Pregnancy, aiming to reduce the adolescent fertility rate by 10% by 2020. The five-pronged strategy involved training health workers, creating adolescent-friendly spaces in primary health centres, promoting a range of contraceptive methods, improving outreach and referrals, and improving school retention and re-entry for pregnant adolescents and adolescent mothers. The strategy was supported by enabling laws and policies, sustained levels of human and financial resources across changing administrations, and intensive collaboration between key stakeholders, including research organizations, civil societies, and youth and women’s advocates. The outcome was an impressive decrease in the adolescent fertility rate from 55 births per 1,000 women aged 15–19 in 2007 to 41 births per 1,000 in 2018.(15) Other countries that have successfully introduced strategies to reduce adolescent pregnancies include Ethiopia, Jamaica, and the United Kingdom.(27).

Another contributor to preterm birth rates is infertility treatments. Recent WHO estimates suggest that one in six adults worldwide experienced infertility in 2022 [39, 40]. Data from some higher-income countries where age at first birth is increasing (advanced maternal age is a known risk factor for reduced fecundity and for preterm birth) suggest that overuse of multi-embryo transfers for in vitro fertilization (IVF) are contributing to higher preterm birth rates [41, 42]. As access to infertility treatments expands, IVF could become a more substantial contributor to preterm birth rates unless countries adopt recommended IVF policies such as single embryo transfer that reduce the risk of multiple pregnancies [43].

Improving the nutritional status of women and girls before pregnancy and during inter-pregnancy intervals could also reduce the risk of preterm birth, particularly policies that address food insecurity and increase access to a balanced diet [44]. More research is needed on the benefits of supplementation on preterm birth.

### Package 2: High-quality antenatal care to prevent preterm birth.

High-quality antenatal care is vital for ensuring a healthy pregnancy and for identifying women and adolescents at increased risk of adverse pregnancy outcomes including preterm birth as well as for the provision of effective interventions to mitigate and manage risks. Since 2016, WHO has recommended that pregnant women and adolescents should have a minimum of eight antenatal care contacts [17]. An underlying driver for whether women receive eight or more antenatal contacts is the timing of the first visit, which should occur prior to 12 weeks’ gestation. In many LMICs, few women receive their first ANC visit in the first trimester. Interventions tailored to different contexts that can improve early antenatal care participation, including community-based interventions targeting social norms, need to be developed and rolled out [45]. All women should have a dating ultrasound prior to 24 weeks gestation, which is critical because accurate gestational age estimation underpins the safe and timely use of preterm-related interventions [46].

Although coverage of antenatal care has increased over the past decade, there is an urgent need to ensure that antenatal care contacts result in provision of timely, evidence-based care. Many interventions that can be provided during pregnancy such as optimizing maternal diet and nutrition and treating infections have been shown in systematic reviews to prevent preterm birth [47, 48]. The increasing prevalence of obesity, older maternal age, and sedentary lifestyle contribute to the incidence of gestational diabetes and preterm birth, making these risk factors important targets for interventions [50]. Some medications and supplements provided antenatally to prevent the occurrence of certain pregnancy complications have “downstream” benefits for preventing preterm birth; low-dose aspirin for preeclampsia prevention is one such an example [51]. Research has also shown that some interventions previously thought to reduce preterm birth are ineffective, including bedrest and activity restriction, avoidance of vaginal sex, and vitamin D supplementation [52]. Even when the latest evidence is adopted into country and international guidelines, there is often a significant gap between current knowledge and common clinical practice that warrants attention to improve uptake.

How care is delivered during antenatal visits is important for positive obstetric outcomes for both mother and baby. The value of person-specific, woman-centred antenatal care is increasingly being recognized, as is the need for tailored management of risk factors for preterm birth [3]. For example, more frequent monitoring is recommended for women with a multiple pregnancy. Some pregnant women need support related to gender-based violence, recreational drug use, healthy weight, or exposure to environmental pollution [53–56]. All women should be screened for mental health issues during antenatal care visits. Common perinatal mental disorders (e.g., depression, anxiety, and somatic disorders) are a risk factor for preterm birth and a major cause of disability. Almost one in every five women in LMICs experiences one or more mental health issues during pregnancy or after childbirth [58]. These mental health disorders can, in turn, affect the well-being of the baby with short and potentially long-term effects.

Some health system arrangements may also reduce preterm birth rates. Group antenatal care, where women participate in pregnancy-related educational and peer support activities with other women, has been shown to increase antenatal care continuation and coverage of some interventions that may decrease the risk of preterm birth (e.g., intermittent preventive treatment of malaria in pregnancy) [59]. Further implementation research is needed to better understand the effectiveness of group antenatal care on preterm birth across different contexts and settings. By utilizing antenatal predictive clinical tools like sonographic cervical length measurement and other biomarker testing, clinicians can better assess a woman’s risk profile and implement appropriate preventive measures. For instance, asymptomatic women with a cervical length ≤ 25 mm detected via ultrasound may be candidates for vaginal progesterone, which has been shown to reduce the risk of preterm birth. Similarly, those with a history of preterm birth and significant cervical shortening may benefit from cerclage placement to prolong pregnancy and improve outcomes [33].

### Package 3: Care around the time of birth, including for women experiencing preterm birth

As shown in Fig. 2, women worldwide are increasingly giving birth in facilities. However, as with antenatal care, the coverage and quality of intrapartum care varies. Improving the safety and quality of intrapartum care is critically important for managing preterm deliveries, reducing maternal and newborn deaths, and for preventing stillbirths, 40% of which happen during labour [20]. The overwhelming majority of intrapartum stillbirths are preventable with high quality childbirth services provided according to standards. However, many facilities lack adequate numbers of staff to monitor women in labour and provide them with recommended interventions in a timely fashion.

Most preterm births involve no known risk factors. When preterm labour or preterm prelabour rupture of the membranes (PPROM) takes place, women need tertiary preventive care (Table 1). The most critical element of this care is a course of antenatal corticosteroid (ACS) injections such as intramuscular dexamethasone or betamethasone. When given to women with a high likelihood of birth prior to 34 weeks’ gestation, it reduces the risk of neonatal mortality and speeds up fetal lung development so that a preterm baby can breathe more easily at birth [60]. WHO guidelines support the use of antenatal corticosteroids (ACS) and tocolytics in low-resource settings, emphasizing their potential to improve neonatal outcomes when used appropriately. Tocolytics alone do not seem to improve newborn health outcomes, though they can provide a longer interval for antenatal corticosteroids to take effect [61, 62]. However, WHO stresses that these interventions should be implemented with proper screening, monitoring, and access to supportive care to maximize benefits and minimize risks. In the past decade, the safe and appropriate use of ACS in LMICs has been actively researched. According to current recommendations, ACS should be used in women prior to 34 weeks’ gestation where preterm birth is considered imminent, and no maternal infection is present (Table 2) [64]. Recent trials in high-resource settings suggest there may be a beneficial role for ACS after 34 weeks’ gestation; a WHO-led trial is currently exploring this application in low resource countries [61]. However, ACS should only be used where adequate childbirth and preterm newborn care are available. Inappropriate use of ACS, particularly when a full-term baby is exposed, or a “just in case” strategy may cause long-term harm and should be avoided [65, 66].

**Table 1:** Definitions of primary, secondary and tertiary prevention of preterm birth [3, 4]

Concept	Definition
Preterm birth	A live born baby before 37 completed weeks of gestation
Risk factors for preterm delivery	Maternal age, low or high body mass index, smoking, maternal stress and socioeconomic factors as education, poverty and social factors. There are also factors related to women’s former obstetrical history as previous preterm delivery, cervical factors such as conisation and uterine malformations. Factors related to current pregnancy is multiple gestation, fetal malformations, uterine bleeding, infections and maternal condition as maternal intercurrent diseases (diabetes, rheumatic diseases and conditions like hypertensive diseases and preeclampsia) [3]
Primary prevention	Interventions for all pregnant women
Secondary prevention	Interventions for women identified as having a specific risk factor. Identifying women more likely to give birth preterm involves undertaking a thorough history, clinical examination, and often a predictive test such as cervical length measurement using ultrasound or other biomarker testing. Women identified as high-risk can be offered therapies, such as progesterone (short cervix) or cerclage
Tertiary prevention	Interventions made after preterm labour has commenced, primarily intended to improve the health of preterm newborns in early life

**Table 2:** The safe and appropriate use of antenatal corticosteroids for preterm birth: Cambodia and the Philippines

The Antenatal Corticosteroids Trial, an implementation trial in six LMICs, found that efforts to scale up antenatal corticosteroids (ACS) in limited-resource settings could harm mothers and babies. This finding led WHO to conduct the Antenatal Corticosteroids for Improving Outcomes in Preterm Newborns (ACTION)-I trial to determine whether and how ACS could safely be used in low-resource countries [99]. The trial found that a course of intramuscular dexamethasone reduced neonatal death and caused no maternal nor newborn harm. In 2022, WHO affirmed its recommendation that ACS therapy be used for women at risk of preterm birth from 24 to 34 weeks of gestation when the following five criteria are met [97].
1. Gestational age assessment can be accurately undertaken and number of babies per delivery is counted
2. There is a high likelihood of preterm birth within seven days of starting therapy
3. There is no clinical evidence of maternal infection
4. Adequate childbirth care is available, including the capacity to recognize and safely manage preterm labour and birth
5. The preterm newborn can receive adequate care, including resuscitation, kangaroo mother care, thermal care, feeding support, infection treatment and respiratory support, including continuous positive airway pressure as needed
*Improving uptake of ACS in Cambodia and the Philippines*
The first Born Too Soon report highlighted generally high rates of preterm birth in Cambodia (10.5%) and the Philippines (14.9%) [98, 100]. Despite prioritizing the reduction of mortality from preterm birth and including the use of ACS in both countries’ national guidelines, uptake of ACS remained low. A situational analysis was undertaken to identify the key barriers to the implementation of ACS. Stockouts were rare and cost was not reported to be a barrier because mothers were not charged for the medication. However, data from both settings identified poor provider knowledge about implementing ACS effectively as a key challenge
In collaboration with the Ministries of Health of Cambodia and the Philippines, a non-randomized study was conducted in 2013–2014. A standardized one-day technical training course on preterm birth and ACS use was delivered to clinical managers and skilled birth attendants in 12 facilities across Cambodia and the Philippines, followed by a monthly audit and feedback process. Monitoring and evaluation of this work identified that coverage of at least one dose of ACS (dexamethasone) increased from 35% at baseline to 86% at endline in Cambodia, and from 34% at baseline to 56% at endline in the Philippines, among women who gave birth at 24–36 weeks’ gestation. This encouraging study shows that is it possible to rapidly overcome lack of provider knowledge and increase coverage of ACS for women at risk of preterm birth. The study also showed that gathering information about the key barriers in the specific subnational context is essential to inform appropriate action

While administering ACS to women with a high risk of early preterm birth is standard practice in high-income countries, its use in LMICs is suboptimal [67]. Multi-country analyses have identified health system bottlenecks that can hinder the uptake of ACS in the health system, including lack of clear appropriate guidelines and programmatic work to strengthen the supply chain managements of essential medicines, inadequacies in leadership, governance and training, health service delivery, health financing, health information systems and technologies [68, 69]. However, there are encouraging examples of LMICs that are overcoming barriers to the appropriate use of ACS (Table 2).

Tocolytic drugs can slow or arrest preterm labour, allowing more time both for ACS to work and to transfer the woman to a higher-level care facility if needed [62]. In 2022, WHO updated its guidelines to recommend using nifedipine as the first-line option for tocolysis when spontaneous preterm labour is present [62]. Tertiary prevention care should also include magnesium sulphate for women likely to give birth before 32 weeks’ gestation, to reduce the risk of cerebral palsy. Also, when PPROM is present, administration of specific antibiotics can prolong pregnancy and prevent neonatal morbidities. There are considerable benefits in reducing mortality and morbidity with delayed cord clamping in prem babies. Like full-term babies, preterm babies also have a pronounced effect of early initiation of breastfeeding [70, 71]. Kangaroo mother care is another effective intervention that should be started as soon as possible after childbirth [72]. Implementation of such strategies would be very efficient and relatively easy through education.

Caesarean section is an essential lifesaving surgical procedure and is performed when vaginal birth is not possible or when it poses a risk to the health of mother and baby. Caesarean section rates worldwide have steadily increased over the past 30 years, and in some countries, they account for more than half of all births (e.g., Dominican Republic (58%), Brazil (56%), Cyprus (55%)) [73]. Worldwide, the mode of delivery for approximately one in five births is now by caesarean section and this ratio is projected to increase to nearly 30% (close to one in 3) by 2030 [73, 74]. Caesarean section delivery can also increase the risk of a preterm birth in a subsequent pregnancy. Increasing caesarean section rates are a worrying trend that will have consequences for preterm birth rates if not addressed appropriately [75]. There is evidence that the widespread practice of inducing labour and performing caesarean sections without medical indications, for example, is contributing to a rise in avoidable preterm births [76]. The drivers of caesarean section delivery are, however, complex and include health system structural factors, health professional practices, and women’s and families’ personal and cultural preferences [77]. Reducing financial incentives to perform caesareans, informing women about mode of delivery options and the risks of non-medically indicated caesarean sections, offering midwifery continuity of care, and changing provider practices around labour induction and scheduling deliveries are some approaches that can slightly counter the escalating rise in caesarean section deliveries [74, 78, 79].

### Package 4: Postnatal care for women following preterm birth

The postnatal period – the first six weeks after birth – is a critical time for newborn and maternal survival, and for supporting both the newborn’s healthy development and the woman’s mental and physical recovery and well-being. Worldwide, more than 3 in 10 women and newborns do not receive postnatal care in the first days after birth, when most maternal and newborn deaths occur [16]. In 2022, WHO published new guidelines on routine postnatal care for women and newborns [16] and updated its guidelines on the care of preterm and low birth weight infants [80]. The guidelines emphasize the importance of good-quality, respectful postnatal care, with a minimum of four postnatal contacts, and ensuring that all women are provided with contraceptive information and services [16].

Even with such support, women with preterm infants are at greater risk of postnatal depression, requiring additional care and support. In cases of preterm birth, women and families should be involved in the routine care of their babies while their babies are still receiving treatment in health-care facilities, which has been shown to improve the experience of women and families, improve health outcomes for the baby, and decrease parental anxiety. Women and families of preterm infants describe the importance of being actively involved in their baby’s care, needing emotional and logistical support and clearly communicated information, and valuing positive relationships with compassionate and respectful health-care providers [81]. Parental leave and entitlements are necessary to address the special needs of mothers, fathers, and other primary caregivers of preterm and other vulnerable newborns.

Every stillbirth and death of a newborn baby, including deaths among babies born too soon, is a tragedy and has a devastating impact on bereaved parents and families who have higher rates of depression and anxiety yet often do not receive the care and support they need [82, 83]. Best practice guidelines recommend that parents should be offered the opportunity to see and hold a stillborn baby, and many studies suggest that parents can benefit from spending time with their stillborn baby in a supportive environment. However, these practices are often not followed, compounding the grief and trauma experienced by bereaved families. There are inspiring examples of bereavement care such as the Birth with Dignity project founded in Uganda in 2017 by nurses. Birth With Dignity has trained hundreds of Ugandan nursing students, midwives, and physicians on high-risk perinatal care and bereavement care. The training includes educating mothers during labour on the grief process and the importance of seeing and holding their baby if it is stillborn or dies soon after birth. Bereavement models and training for health-care professionals on how to provide bereavement care should include stillbirths and newborn and maternal deaths, addressing both the needs of families and the adverse consequences for health-care providers. Guidelines have been developed for health care professionals and by advocacy organizations that outline steps to follow [84–86].

### What is needed to improve service delivery?

The programmatic priorities listed above all rely on well-functioning health systems, with maternal and newborn care as a foundation of universal health coverage (UHC), including primary health care. The factors listed below are key to the successful and sustainable implementation of high-quality maternal healthcare services.

Investments in human resources and staffing – including in educational curriculums, regular training opportunities, and in equipment and supplies – is essential for ensuring enough well trained, competent, and motivated health-care providers are available, equitably distributed, and placed in enabling environments [28]. The global shortage of healthcare providers is particularly severe in LMICs [88]. Many maternity services are understaffed, compromising patient safety, and limiting the quality of care. Health-care providers also need high-quality pre- and in-service education and refresher training, managerial and clinical leadership, and a contemporary scope of practice that ensures they can deliver high-quality, evidence-based preterm care according to standards. They also need written, up-to-date clinical protocols to guide their preterm labour prevention and management decisions that are context specific [61, 62, 89].

Maternity health-care providers highly value opportunities to improve their knowledge and skills in preterm birth management [90]. Effective strategies include drills, simulation exercises, supportive supervision, on-the-job training and mentoring, as well as continuous quality improvement and multidisciplinary care models for managing preterm labour [90, 91]. Where new care models are introduced, such as group antenatal care or midwifery continuity of care, health-care providers require orientation, a facilitative environment for providing care, and mentorship from experienced staff.

Collectively, health-care providers require an enabling and adequately resourced environment. The delivery of good-quality preterm care in low- and middle-income contexts is often restricted by stockouts of essential medicines or supplements, inadequate infrastructure, dysfunctional equipment and outdated restrictions on scope of practice, particularly for nurses and midwives. The quality of essential medicines, including cold storage for ACS, may also be poor in some settings. These issues are best addressed through health system strengthening, including enforcement of strong regulation, and shoring up logistics and supply systems so that health facilities are ready to provide high quality services to all [90].

Gestational age at birth and number of children per birth (e.g., singleton, twin, triplets) should be regularly collected and reported through routine country health information systems [92]. Currently, gestational age is infrequently collected and reported in national registries in most LMICs. System-wide efforts to establish and improve birth registries across facilities are needed, including creating a culture of “good documentation” and using electronic systems to simplify data capture and reporting [93, 94]. Similarly, wide-scale implementation of Maternal and Perinatal Death Surveillance and Response would improve understanding of factors contributing to deaths due to prematurity and ability to develop effective, contextualized preterm birth interventions [95].

Referral systems are critical for ensuring women and newborns receive emergency and higher-level care when needed [16, 96]. Timely and appropriate referral to a higher-level facility is required for women presenting in preterm labour at a lower-level health facility. They may need ACS and tocolysis before a rapid transfer to a higher-level centre where adequate preterm newborn care is available. Referral systems must also ensure that women and preterm newborns are reconnected to their primary and community health-care providers after discharge from higher-level care. Yet referral networks in LMICs, particularly in remote and rural areas, are often inadequate because of poor roads, few ambulances, communication challenges, and long distances between facilities and other factors. Investments are needed to develop, strengthen and sustain referral pathways in LMICS to enable the continuation of service provision during referrals particularly in emergencies [25].

### Pivots

This section describes recommended actions to improve health systems for the prevention and clinical management of preterm births, which will also result in overall improvements in maternal and newborn health and in the reduction of stillbirths.

#### Pivot 1: Government leaders, health professional associations, civil society members, private sector actors, and all development partners must join forces to ensure inclusion of sexual, reproductive, and maternal health services within UHC strategies, including the integration of interventions for known, modifiable risk factors for preterm birth.

As countries define their UHC policies and programmes, effort should be made to ensure equitable, high-quality sexual, reproductive and maternal health services, grounded in evidence are included. These services plus child health services are the core of primary health care and achievement of UHC depends upon their scale-up. Many maternal health services, including interventions to prevent and manage preterm birth, are health-promotive and cost-effective. Improving access to them and their quality will require financial strategies that reduce out-of-pocket expenditures and increase investments in health system readiness [36]. Special efforts are needed to ensure family planning services are available to underserved populations and across the life-course, including postpartum prior to hospital discharge and during adolescence to prevent teenage pregnancies. There is also a need for broader public health education and communication on behavioural and lifestyle changes to prevent preterm birth, such as smoking cessation, improved nutrition, and physical activity for all women and adolescent girls.

#### Pivot 2: Fully leverage existing tools to improve the management of preterm birth, including appropriate use of ACS.

Women at risk of imminent preterm birth need timely, evidence-based interventions to improve the health outcomes for their preterm babies. Compared to a decade ago, there is now stronger evidence of the effectiveness of ACS; however, ACS coverage remain suboptimal in many LMICs. Greater political commitment and investment are needed to ensure that this evidence is prioritized and acted upon. More research is needed to identify barriers to implementation, as well as greater sharing of country learnings on overcoming these barriers to scale up the effective use of these commodities. Improvements in health information systems including civil and vital registration systems and implementation of MPDSR are also critical for tracking the burden of preterm births and understanding and addressing causal mechanisms.

#### Pivot 3: Ensure that women, adolescents, and families receive high quality, respectful, person-centred care and that women’s voices are respected.

A major global shift in maternal and newborn health care over the past decade has been a focus on increasing coverage of *high-quality care* before, during and after pregnancy and childbirth. Across the continuum of care, opportunities must be seized to increase service access and service quality. Each contact with the health-care system is an opportunity to provide women with essential care including preterm birth prevention and management services provided respectfully and according to standards. A plethora of new global policies and guidelines on maternal health care have been developed over the past decade. There is no time to lose in closing the implementation gap and translating these guidelines and policies into provider practice.

Positive experiences are a vital dimension of service quality. Women who received midwife continuity of care report more positive experiences during pregnancy, labour, and the postnatal period [79]. Services that ensure positive experiences include those characterized by effective communication, respect, preservation of dignity, social and emotional support, and listening and responding to the concerns of women and families. Women and their partners may be learning about preterm birth for the first time in a stressful context, such as preterm labour. They need readily understandable information that is provided sensitively about treatments, side-effects and prognoses for their baby and themselves. The need for respectfully provided and easy to understand information can be even greater in the postnatal period. Compassionate bereavement care for women and families who have experienced the death of a baby, whether stillborn or due to complications after birth including a preterm birth, is urgently needed. Beyond provider–client communications, women’s and families’ voices must be heard in broader conversations about perinatal deaths and they should be actively involved in developing the policies and services that affect them. Their voices are a powerful means of increasing social accountability for the delivery of equitable, accessible and high-quality services.

The strategies described for each of these three pivots could, if implemented, save lives and improve the health outcomes of women and newborns who are at risk of or affected by preterm birth. They support the broader Born too Soon agenda by addressing the causes, consequences and solutions of the global challenge of preterm birth, which affects millions of families every year. It is important to capitalize on opportunities to prevent and manage preterm birth through investments in health system strengthening, including ensuring sufficient numbers of skilled providers are trained and equitably deployed, and that they work in environments that enable them to deliver essential services to all women and newborns who need them i.e., facilities with running water and electricity and a consistent supply of medicines and equipment, plus a functioning referral network. Shoring up health systems should also involve development and adoption of innovations that help ensure service continuity in the event of external shocks such as pandemics, climate events, and economic crises. The Born too Soon agenda and the 77 th WHA resolution are a call to action to prioritize the prevention and care of preterm birth as part of efforts to achieve universal health coverage and Sustainable Development Goal 3.

## Conclusion

Ensuring equitable access to high-quality sexual, reproductive, and maternal health services, especially in resource-limited and fragile settings, is pivotal for achieving significant advancements in maternal health and reducing preterm births. Enhanced service accessibility is essential for the successful implementation of the World Health Assembly resolution passed in May 2024, which focuses on accelerating progress towards meeting the SDG targets on maternal, newborn, and child mortality.

To achieve progress on reducing preterm birth, government leaders, civil society members, private sector actors, and all development partners must unite to ensure that sexual, reproductive, and maternal health services are included within universal health coverage strategies. This includes integrating interventions for known modifiable risk factors for preterm birth. It is crucial to fully leverage existing tools to improve the management of preterm birth, such as the appropriate use of ACS. Furthermore, it is essential to provide high-quality, respectful, person-centred care to women, adolescents, and families. Health sector improvements need to be complemented by well-coordinated multi-sectoral strategies that address underlying determinants of women’s and adolescents’ health and increase access to services through other platforms such as the education system.

## Data Availability

All data is available in the paper or in supplementary files. Additional information is available at www.borntoosoonaction.org.

## Abbreviations

ACS: Antenatal corticosteroids
LMIC: Low- and Middle-Income Countries
PPRO: Preterm prelabour of rupture of membranes
SDG: Sustainable Development Goal
UN: United Nations
UNC: Universal health coverage
UNFPA: United Nations Population Fund
UNICEF: United Nations International Children's Emergency Fund
WHO: World Health Organization
